# Victimization, polyvictimization, and depression symptoms among immigrants and native children and youth in Chile

**DOI:** 10.1186/s13034-024-00755-7

**Published:** 2024-06-18

**Authors:** Cristián Pinto-Cortez, Mauricio Marín-Gutiérrez, Carlos Melis-Rivera, Lorena Contreras-Taibo, Rodrigo Moya-Vergara

**Affiliations:** 1 Escuela de Psicología y Filosofía, Universidad de Tarapacá, 18 de Septiembre 2222, Arica, Chile; 2 Facultad de Psicología, Universidad Diego Portales, Grajales 1898, Santiago, Chile; 3 Escuela de Psicología, Universidad Católica del Norte, Angamos 0610, Antofagasta, Chile

**Keywords:** Migration, Polyvictimization, Depression, Children and youth, Chile

## Abstract

**Background:**

Migration exposes children and youth to vulnerabilities, including uprooting, lack of protection, limited access to services, and violence. Previous studies have shown that victimization experiences impact the mental health of migrant children, including depression, anxiety, and post-traumatic stress disorder. This study aims to examine the co-occurrence of multiple forms of maltreatment (polyvictimization) among migrant and Chilean children and youth and its association with depressive symptoms, addressing a research gap in Latin America.

**Methods:**

Secondary data from the National Polyvictimization Survey (NPS) conducted by the Chilean Ministry of the Interior were analyzed. Measures assessing polyvictimization and depressive symptoms were administered to a sample of 1362 participants, with equal group sizes for migrants and Chilean-born individuals. Data analysis included descriptive statistics, group comparisons, correlation analyses, and multiple regression analyses.

**Results:**

The study revealed marked differences in experiences of conventional crime victimization and polyvictimization between migrant and Chilean-born participants, with migrants facing slightly higher incidences. Correlational analysis indicated variable strengths of association between victimization types and depressive symptoms across groups, with Chilean-born individuals showing stronger correlations for certain victimization forms. Multiple regression analysis highlighted gender, polyvictimization, child maltreatment, internet victimization, sexual victimization, and peer/sibling victimization as significant predictors of depressive symptoms across the sample. Notably, an interaction was observed between child maltreatment and migrant status, indicating a mitigated impact of maltreatment on depressive symptoms among migrant adolescents. This suggests the potential for unique resilience or coping mechanisms in this group.

**Conclusions:**

This study elucidates the varied victimization experiences of migrant children and youth in Chile, with a notable emphasis on the mitigating effect of migrant status on the relationship between child maltreatment and depressive symptoms. It highlights the resilience and potential adaptive strategies of migrant minors facing adversity. The findings underscore the necessity of developing support and intervention strategies that recognize the specific needs and strengths of migrant children and youth, advocating for policies that protect and empower this vulnerable demographic amidst new environmental challenges.

## Introduction

Migration has a wide range of definitions. Currently, the most accepted one states that migration is the change of residence involving crossing a defined geographical, national, or international boundary. In this context, we define international migration as movement between nations. If the boundaries crossed correspond to a recognized demarcation within a country (between governmental divisions, and urban and rural areas, among others), migration is called internal migration [1].

Migration is driven by various factors, from humanitarian reasons to socioeconomic challenges [2]. Currently, individuals of all ages migrate across diverse regions, facing climatic, humanitarian, and social adversities that can pose risks to their lives. Given this context, children and adolescent migrants are a particularly vulnerable group due to their evolutionary stages, as the adversities they are exposed to during their transit can significantly impact their psychological and social development. Thus, migrant youth may be affected by uprooting, lack of protection, lack of access to health and education, violation of their rights, and exposure to victimization by violence and abuse [3].

Addressing this issue, Jud et al. [4] conducted a systematic review of 17 studies on the prevalence of violence affecting migrant children. The finding showed high variability in the data, especially in the statistics related to physical (9-65%) and sexual abuse (5-20%). This review documented that refugee and migrant children presented at least one type of abuse (physical maltreatment, physical violence, sexual abuse, neglect, or other traumatic experiences) throughout their lives [4]. It should be emphasized that of the 17 studies reviewed, most of the research focused on evaluating a specific type of violence or an adverse or traumatic experience. No approach to the co-occurrence of maltreatment or exposure to multiple types of violence was adopted in these studies. Additionally, it was found that most of the studies conducted in Europe reported extremely high rates of physical abuse of migrant children and adolescents, more than any other geographical areas studied (such as North America or Canada). For South America and Oceania, hardly any studies have been carried out.

In the case of Latin America, victimization and polyvictimization have been studied in different countries in community samples. These studies define polyvictimization as experiences of multiple victimizations such as sexual abuse, physical abuse, bullying, and exposure to family violence in different contexts. In addition, victimization and polyvictimization have been addressed by countries concerning adolescent well-being, but not with the migrant population [5–7].

Another relevant factor to consider is the effect of victimization experiences on the mental health of migrant children and adolescents, which has been made clear by research done on this topic by Craig et al. [8]; Chan et al. [9]. Furthermore, some authors have reported the risk of suffering mental health problems in specific subgroups of migrants, for example, those who have had victimization experiences due to war or severe social conflicts [8]. Similarly, a study on victimization with Latino and Asian migrant youth in the United States found that first-generation migrant students presented high levels of fear and were at greater risk of victimization at school [10]. Other studies have also established that interpersonal victimization and mental health problems are strongly related to immigration status in various countries [11–13]. These problems are mainly related to depression, suicidal thoughts, and anxiety [14, 15], but also to other mental health problems such as post-traumatic stress disorder, aggressive behaviors, and psychosomatic problems [16–18].

While victimization is not always the main factor underlying the mental health problems of migrant children, evidence is increasingly accumulating regarding the predominant role of victimization in the mental health of this specific group [19]. However, despite the studies described above, it is possible to identify a deficit of research regarding migrant children and their mental health needs. This includes factors that could be the basis of these problems, such as interpersonal violence victimization processes. Additionally, the studies conducted on this topic are varied in terms of methodology and the contexts in which they have been developed. However, what they share is their focus on assessing a specific type of victimization and specific mental health problems. Therefore, amassing studies on the relationship between victimization, migration status, and mental health of children and adolescents in different sociocultural contexts and geographical areas are still relevant. A comprehensive approach to child victimization should also be considered, incorporating the assumption that children and adolescents may experience polyvictimization throughout their lives. This is related to mental health problems in different ways.

### Victimization, polyvictimization, and mental health of migrant children in the Chilean context

Chile is a country that from its origins to the present day has been affected by migratory influxes [20]. Starting in the mid-1990s, the country began to experience a surge in the number of immigrants from Andean countries. Until then, immigration had been associated with European countries such as Spain, Italy, Germany, and Yugoslavia [21]. Until 2010, Chile received relatively few refugee applications. However, the numbers significantly increased afterward, due to various regional circumstances [22, 23]. By 2017, when the data for this article were collected, migrants accounted for 4.4% of Chile’s population. Of this percentage, 10.6% corresponded to children and adolescents, predominantly from Peru, Argentina, Bolivia, Colombia, and Venezuela [24]. Currently, the percentage of migrants in Chile corresponds to 7.8% of the population, of which 2.5% corresponds to children and adolescents under the age of 19 [25]. The main migratory flows come from Venezuela, Peru, Colombia, Haiti, Bolivia, Argentina, and Ecuador [26], in order from the most to the least number of migrants. In 1990, the Chilean government ratified the International Convention on the Rights of Children and Adolescents. Therefore, the protection of migrant children’s rights is a constant challenge for Chilean migration legislation [27] and the recurring concern is to guarantee respect for their rights and the psychosocial conditions in which they find themselves. Research from Chile has found that the migratory status of children and adolescents is a risk factor for sexual victimization, child abuse, and peer victimization [28–30]. It has also been reported that migrant Children and adolescents in Chile are exposed to high rates of indirect victimization. This involves witnessing riots, shootings, assaults with or without objects, assaults on siblings, exposure to family violence, witnessing murder and robbery in the home [31]. Nonetheless, the 2017 First National Polyvictimization Survey determined that being a migrant child or adolescent neither increased nor decreased the risk of experiencing polyvictimization [32]. In summary, while migrant status in children and adolescents raised the likelihood of experiencing specific types of victimization, it did not affect the chances of encountering polyvictimization across various contexts.

Regarding to the studies about mental health, most research in this area has focused on a broader concept of mental health as a subjective aspect and everyday life in the migration process [33]. At the time of this review, no research in Chile has been found on the prevalence of mental health problems in migrant children and adolescents. In addition, studies related to victimization and polyvictimization and their direct relationship with mental health problems in this group are in an incipient state from a conceptual/qualitative approach [34, 35].

This topic is relevant since it has been established that mental health is a significant problem in Chilean-born children and adolescents and that it is also related to some forms of mistreatment. For instance, a study by Riquelme et al. [36] with a sample of 1558 children and adolescents between 4 and 18 years of age from four cities in Chile established that the most prevalent mental health problems were disruptive disorders (14.6%), nervous disorders (8.3%) and mood disorders (5.1%). They also concluded that Chilean children who suffered physical and psychological abuse were more likely to develop these three mental health problems. Additionally, in adolescents, mood disorders were associated with psychological abuse and sexual abuse.

## The present study

Migratory phenomena are transit processes that involve a high vulnerability for the people who carry them out, especially for children and youth [37]. However, this vulnerability does not end when people arrive in the destination country, rather, it can be aggravated by the different types of victimization in the receiving countries. This could take place within their families, in the community, and the school [14].

Studies on the topic of victimization and mental health of migrant children and adolescents in the world are in an intermediate phase. Most of these initiatives are being developed in North American, European or Asian countries. There is a constant interest in knowing what happens in other geographical areas of the world, as is the case of Chile with migration occurring among Latin American countries. Although the topic of victimization in the context of migration has been explored in other continents, the study of polyvictimization and mental health in migrant children and adolescents is still in its initial phase. In the case of Chile, the prevalence of victimization and polyvictimization of Chilean-born children and adolescents and the impact of these acts of violence on their mental health have been studied, especially in anxiety, post-traumatic [38], or depressive spectrum problems [32]. However, to our knowledge, no studies have been conducted in Chile on the impact of polyvictimization on depressive symptoms in migrant children and youth. Polyvictimization (PV) and depressive symptoms have only been analyzed in Chilean children and youth, detecting a robust relationship between both variables [30]. Thus, this study aims to examine the experiences of polyvictimization and its relationship with depressive symptoms in migrant children born abroad but residing in Chile to compare them with their Chilean peers. The following research questions are addressed: (1) What are the differences in the types of victimization among migrant children and youth and Chilean-born children and youth; (2) How do various types of victimization affect depressive symptoms based on individuals’ migratory status; (3) How does polyvictimization predict depressive symptoms among migrant children and youth and Chilean-born children and youth. Based on previous studies [32, 39], we hypothesize (H1) a positive association between victimization, polyvictimization, and depressive symptoms. However, as documented by Segura et al. [40] (H2), this hypothesized association will be stronger in immigrant children and youth than in Chilean-born children and youth.

## Methods

### Participants

This study analyzes secondary data from the National Polyvictimization Survey (NPS), conducted by the Chilean “Ministerio del Interior” (Ministry of Interior) through the “Subsecretaría de Prevención del Delito” (Undersecretariat for Crime Prevention). The NPS provided data representative of Chilean children and youth based on the 2016 Ministry of Education Directory of Enrollments and Educational Establishments [32]. The sample selection process was stratified probabilistic and tri-stage, considering (1) educational establishments, (2) grade levels, and (3) enrolled students within each grade. The final sample consisted of 19,684 children and youth aged 12 to 19, drawn from 699 educational establishments (including public, private, and subsidized schools) representing all fifteen regions of the country. The sampling error was ± 0.7%, with a maximum variance, and a 95% confidence interval (CI) was used [32]. Considering the study’s objectives, 212 cases with missing data in the “migration status” variable were excluded. Subsequently, a rigorous exact matching procedure was conducted using an initial sample of 19,436 participants (96.4% native Chileans and only 3.5% migrants). The exact matching aimed to create equivalent groups based on three covariates: gender, age, and grade level to control for confounding and obtain more precise estimates in subsequent analyses. As a result, a final sample of 1,362 participants was obtained, divided into two equal-sized groups (681 natives and 681 migrants), comparable in terms of gender (51% males and 49% females), educational grade distribution (27.5% seventh grade, 23.5% eighth grade, 15.6% ninth grade, 18.6% tenth grade, and 14.8% eleventh grade^1^), and age (14.58 ± 1.62). Additional tests, including a chi-square test for gender (χ^2^(1) = 0.000, *p* = 1.000), and independent samples t-tests for age (t(1360) = 0.000, *p* = 1.000) and grade level (t(1360) = 0.000, *p* = 1.000), revealed no significant associations with migration status. These results support the effectiveness of the matching procedure.

## Measures

### Polyvictimization

A Chilean adaptation of the Juvenile Victimization Questionnaire (JVQ) [41] was used. The adapted questionnaire has 32 items that assess different forms of victimization in children and adolescents, which are grouped into six modules: victimization by conventional crimes, victimization by caregivers, victimization by peers or siblings, sexual victimization, indirect victimization, and electronic victimization. An example item is: “In your lifetime, has a child or adolescent ever hit or physically attacked you?“. The JVQ has a dichotomous response format for victimization (each item is answered with a yes or no) and a continuous score for polyvictimization (sum of all positive responses). The instrument has demonstrated adequate psychometric properties in validation studies in the United States [41], China [42], and Spain [43]. The internal consistency measured by Cronbach’s alpha for the instrument was 0.85, whereas for the modules, the coefficient ranged between 0.48 for electronic victimization and 0.64 for common crime victimization. These values are expected since the items aim to measure life experiences rather than a psychological construct and are consistent with those previously reported for the original version of the JVQ in the child population [41].

### Depressive symptoms

The revised version of the Depression Self-Rating Scale (DSRS-R) [44] developed from the Chilean adaptation of the Depression Self-Rating Scale (DSRS) [45] was used. The DSRS-R is an instrument adapted to the Chilean context and composed of 15 items to assess the presence of depressive symptomatology in children and adolescents. An example of an item is: “I feel very lonely”. It has a three-alternative response format (always, sometimes, and never) with scores ranging from 0 to 2. Ten of its items must be recorded before calculating the total score of the instrument, whose maximum is 30 points. The DSRS-R demonstrated adequate reliability indices (α = 0.917) in a national validation study with the Chilean school population [44].

### Procedure

The study’s methodology, participant demographics, utilized instruments, and data collection protocols for the National Survey of Polyvictimization (NSP) are comprehensively outlined in the Final Report by the National Council for Children [32]. All NSP data were collected during the last quarter of 2017. Following this, the datasets were made accessible by governmental agencies for public utilization, with a specific emphasis on their secondary use by academic institutions within the State of Chile.

### Data analysis

First, descriptive analyses were performed by the group for each variable (e.g., types of victimization, polyvictimization, and depressive symptoms). Since formal normality tests (e.g., Shapiro-Wilk and Kolmogorov-Smirnov) can be unreliable for large samples, skewness, and kurtosis values between − 2 and + 2 were considered, demonstrating the univariate normality of each distribution [46]. Meanwhile, the homoscedasticity assumption was assessed using Levene’s test, where a p-value < 0.05 indicates that the null hypothesis of equality of variances should be rejected. Considering both criteria, subsequent mean comparison analyses between these groups of identical size were performed with Student’s t-test for all variables that met the assumptions for normality and homoscedasticity. Mann-Whitney U test was used for those that did not meet at least one of these assumptions.

Cohen’s d (for the t-test) and Hedge’s g (for the U-test) were used to measure effect size, with values between 0.2 and 0.5 indicating mild effects, 0.5–0.8 moderate effects, and > 0.8 high effects. The association between types of victimization, polyvictimization, and depressive symptoms was analyzed using Pearson’s r-statistic and Spearman’s rho. The z-statistic was calculated to compare correlations between groups according to their migration status. A p-value < 0.05 indicates that the two correlation coefficients are significantly different. Finally, a series of multiple regression analyses (MRA) were performed using the forced-entry method to assess whether the predictive power of victimization on adolescents’ depressive symptoms varies as a function of adolescents’ migration status. To address the variable of migratory status in our analyses, a dummy variable was created. This dummy variable was defined as follows: a value of 0 was assigned to native participants (i.e., those born in Chile), and a value of 1 was assigned to immigrant participants, who were born abroad but reside in Chile. For the MRA, the Durbin-Watson (D-W) statistic was used to assess the assumption of independence of the errors, where values between 1.5 and 2.5 allowed us to assume independence. Meanwhile, the assumption of non-collinearity was tested using the tolerance statistic (T) and the variance inflation factor (VIF), where values > 0.10 and < 10, respectively, imply the assumption of non-collinearity. All statistical analyses were performed using SPSS statistical software version 26.

## Results

### Descriptive statistics and mean comparison

Table 1 presents the descriptive statistics (means and standard deviation) of the measures of the different types of victimization, polyvictimization, and depressive symptoms for each group. It also presents the results of the mean contrasts of these variables according to the migratory status of the participants (Chilean-born children, youth and migrants). The skewness (γ1) and kurtosis (κ) values of the variables analyzed were within the parameters of normality (cut-off score ± 2), except for sexual victimization (γ1 = 3.04 and κ = 11.07). The conventional crime victimization (F = 4.992; t (_1360_)= − 3.060; *p* =.026) and sexual victimization (F = 11.645; t = − 2.156; *p* =.001) variables obtained statistically significant results in Levene’s test, rejecting the null hypothesis of equality of variances.

**Table 1:** Descriptive Statistics and Mean Contrast by Migratory Status

	Native	Immigrant	t (U)	*p*	d (g)
	M (SD)	M (SD)			
**CC**	**2.01 (1.74)**	**2.32 (1.84)**	**(161,406)**	**0.003**	(0.173)
CM	0.83 (1.00)	0.88 (1.04)	− 0.814	0.416	0.049
PSV	1.09 (1.16)	1.08 (1.14)	0.132	0.895	0.008
SV	0.42 (0.91)	0.54 (1.11)	(180,801)	0.091	(0.118)
WIV	2.28 (1.75)	2.42 (1.78)	− 1.411	0.158	0.079
IV	0.40 (0.66)	0.36 (0.63)	1.089	0.276	0.061
**PV**	**6.78 (5.35)**	**7.39 (5.37)**	**− 2.081**	**0.038**	0.113
DS	9.41 (5.68)	9.78 (5.67)	− 1.157	0.248	0.065

The values in bold indicate statistically significant differences between groups (p <.05)

CC = Conventional Crime; CM = Child Maltreatment; PSV = Peer and Sibling Victimization; SV = Sexual Victimization; WIV = Witnessing and Indirect Victimization; IV = Internet Victimization; PV = Poly-victimization; DS = Depressive Symptoms; t = Student’s t test for independent samples; U = Mann-Whitney U test; d = Cohen’s d; g = Hedges’ g

Statistically significant differences (*p* <.05) were identified in levels of victimization for conventional crimes (U = 161,406, *p* =.003) and poly-victimization (t = − 2.081, *p* =.038). The migrant group exhibited slightly higher scores compared to their Chilean-born counterparts (see Table 1). However, it is important to note that the effect size for these differences was relatively small in both cases (Cohen’s d and Hedges’ g were less than 0.2).

### Correlation between variables according to migratory status

A significant direct relationship was found between all the variables under study, regardless of the group to which they belonged^2^. Notably, moderate to strong direct associations were observed between each type of victimization and polyvictimization (r/rho Chilean born children and youth between 0.541 and 0.842, *p* <.001; r/rho migrants between 0.542 and 0.810, *p* <.001). Slight direct relationships between all forms of victimization (including polyvictimization) and depressive symptoms (r/rho Chilean born children and youth between 0.245 and 0.406, *p* <.001; r/rho migrants between 0.228 and 0.364, *p* <.001) were found.

There were differences when comparing the correlations obtained between the groups, highlighting higher coefficients in the Chilean-born children and youth group for depressive symptoms with victimization by conventional crimes (rho = 0.274, *p* <.001), victimization by caregivers (*r* =.406, *p* <.001), victimization by peers (*r* =.283, p <. 001), indirect victimization (*r* =.245, *p* <.001), electronic victimization (*r* =.318, *p* <.001), and polyvictimization (*r* =.405, *p* <.001). Meanwhile, the relationship between depressive symptoms and sexual victimization (rho = 0.283, *p* <.001) was found to be slightly higher in the migrant groups. However, the z-contrasts indicated that the differences in the correlations of all variables were not significant according to the migratory status of the participants (*p* >.05 in all z-contrasts).

### Sociodemographic factors and polyvictimization as predictors of depressive symptoms

To explore how sociodemographic factors and polyvictimization predict depressive symptoms in adolescents, we conducted three multiple regression models using the forced-entry method (see Table 2). The first model demonstrated significant explanatory capability for the variance in depressive symptoms (F_[8, 1342]_ = 39.119, *p* <.001, D-W = 2.030), achieving an adjusted R² of 0.183. Among the sociodemographic variables included in the regression, gender stood out as a significant predictor of depressive symptoms (β = 0.193, t = 7.834, *p* <.001), indicating that female adolescents tend to exhibit more depressive symptoms than their male counterparts. The analysis further revealed that living with certain family members did not significantly impact depressive symptoms; however, residing with the father exhibited a notably negative association (β = − 0.065, t = − 2.513, *p* =.012), suggesting the father’s presence may have a protective effect against the development of depressive symptoms. Presence of a disability was also a significant predictor (β = 0.099, t = 4.045, *p* <.001), highlighting the vulnerability of this group among adolescents. Nonetheless, the most robust predictor was polyvictimization, which showed a positive relationship (β = 0.346, t = 13.957, *p* <.001), indicating that higher exposure to various forms of victimization is associated with increased levels of reported depressive symptoms.

**Table 2:** Multiple Regression Models of Sociodemographic Factors and Polyvictimization as Predictors of Depressive Symptoms

Predictors	Model 1					Model 2					Model 3				
	B	SE_B_	β	t	*p*	B	SE_B_	β	t	*p*	B	SE_B_	β	t	*p*
*Constant*	**6.977**	**0.524**		**13.307**	**0.000**	**6.356**	**0.819**		**7.758**	**0.000**	**6.616**	**0.648**		**10.213**	**0.000**
Gender	**2.093**	**0.267**	**0.193**	**7.843**	**0.000**	**2.232**	**3.78**	**0.206**	**5.909**	**0.000**	**2.242**	**0.377**	**0.207**	**5.942**	**0.000**
Mother	− 0.709	0.467	− 0.039	-1.518	0.129	− 0.425	0.748	− 0.023	− 0.568	0.570	− 0.725	0.472	− 0.039	− 0.1538	0.124
Father	**− 0.712**	**0.283**	**− 0.065**	**-2.513**	**0.012**	**− 0.939**	**0.413**	**− 0.086**	**− 2.272**	**0.023**	**− 0.944**	**0.413**	**− 0.086**	**− 2.288**	**0.022**
Siblings	− 0.108	0.299	− 0.009	− 0.361	0.718	0.103	0.434	0.009	0.237	0.812	0.135	0.429	0.012	0.315	0.753
Grandparents	− 0.081	0.375	− 0.005	− 0.217	0.828	0.524	0.506	0.035	1.035	0.301	0.492	0.502	0.033	0.980	0.327
Other relatives	− 0.071	0.386	− 0.005	− 0.184	0.854	0.273	0.612	0.018	0.446	0.656	0.269	0.612	0.018	0.439	0.661
Disability	**1.399**	**0.346**	**0.099**	**4.045**	**0.000**	**1.130**	**0.513**	**0.080**	**2.205**	**0.028**	**1.145**	**0.512**	**0.081**	**2.239**	**0.025**
PV	**0.349**	**0.025**	**0.346**	**13.957**	**0.000**	**0.375**	**0.036**	**0.371**	**10.513**	**0.000**	**0.375**	**0.036**	**0.371**	**10.515**	**0.000**
Migrant	–	–	–	–	–	1.135	1.077	0.105	1.054	0.292	0.713	0.705	0.066	1.012	0.312
Gender*Migrant	–	–	–	–	–	− 0.276	0.535	− 0.022	− 0.517	0.605	− 0.280	0.534	− 0.022	− 0.524	0.600
Mother*Migrant	–	–	–	–	–	− 0.499	0.964	− 0.406	− 0.518	0.605	–	–	–	–	–
Father*Migrant	–	–	–	–	–	0.475	0.570	0.038	0.834	0.405	0.476	0.570	0.038	0.835	0.404
Siblings*Migrant	–	–	–	–	–	− 0.348	0.600	− 0.030	− 0.580	0.562	− 0.413	0.586	− 0.035	− 0.704	0.482
Grandparents*Migrant	–	–	–	–	–	**-1.577**	**0.777**	**− 0.068**	**− 2.029**	**0.043**	**− 0.1537**	**0.773**	**− 0.067**	**− 1.987**	**0.047**
Other relatives*Migrant	–	–	–	–	–	− 0.644	0.793	− 0.034	− 0.812	0.417	− 0.619	0.791	− 0.033	− 0.783	0.434
Disability*Migrant	–	–	–	–	–	0.487	0.696	0.027	0.699	0.484	0.477	0.696	0.026	0.686	0.493
PV*Migrant	–	–	–	–	–	− 0.051	0.050	− 0.049	− 0.1009	0.313	− 0.050	0.050	− 0.049	− 0.1002	0.316
Model Fit			F	**39.119**	**0.000**			F	**18.946**	**0.000**			F	**20.125**	**0.000**
			R^2^	**0.188**				R^2^	**0.193**				R^2^	**0.193**	
			R^2^_adj_	**0.183**				R^2^_adj_	**0.183**				R^2^_adj_	**0.184**	

The values in bold indicate statistically significant values (p <.05)

PV = Polyvictimization; β = Standardized Beta

Models 2 and 3 (as shown in Table 2) incorporated a migrant dummy variable and interaction effects between the rest of the independent variables and the dummy variable. This approach was employed to assess the differential impact of predictors on depressive symptoms between migrant and non-migrant adolescents. The inclusion of the migrant dummy and interaction terms allows for a nuanced understanding of how migration status may moderate the relationships between predictors and depressive symptoms. Model 2 accounted for the same proportion of variance in depressive symptoms as Model 1, achieving an adjusted R² of 0.183 (F_[17, 1344]_ = 18.946, *p* <.001, D-W = 2.037). Similarly, gender, living with the father, having a disability, and polyvictimization maintained their significant roles and directions of influence on depressive symptoms. However, the results reveal that migrant status itself, as well as most interactions between this status and other independent variables, did not significantly impact depressive symptoms. This suggests that being a migrant, by itself, does not significantly increase or decrease the risk of experiencing depressive symptoms compared to non-migrant adolescents. However, a notable exception was observed in the interaction between being a migrant and living with grandparents, indicating a significant protective effect (β = − 0.068, t = − 2.029, *p* =.043). This could be interpreted as living with grandparents providing a unique form of support or stability that could help to mitigate depressive symptoms in migrant adolescents. Concerning the interaction between living with the mother and being a migrant, a variance inflation factor (VIF) exceeding the cutoff score was observed (VIF = 12.975). This suggests potential multicollinearity issues, implying redundancy that might distort the outcomes of the regression analysis. Due to this, a third model was conducted excluding this variable to ensure the stability and reliability of the findings. Model 3 showed a slight improvement over the previous model, significantly explaining the variance in depressive symptoms (F_[16, 1345]_ = 20.125, *p* <.001, D-W = 2.037), with an adjusted R² of 0.184. Once again, predictors such as gender, living with the father, having a disability, polyvictimization, and the grandparents*migrant interaction maintained their significance. It is also worth noting that polyvictimization was the predictor with the most weight on depressive symptoms across all three models.

### Sociodemographic factors and types of victimization as predictors of depressive symptoms

We conducted three multiple regression models to determine how sociodemographic factors (such as gender, living arrangements, and the presence of a disability) and specific types of victimization predict depressive symptoms in adolescents (refer to Table 3). The initial model (Model 4 significantly accounted for variations in depressive symptoms, achieving an adjusted R² of 0.199 (F_[13, 1348]_ = 27.048, *p* <.001, D-W = 2.050). Gender (β = 0.160, t = 6.353, *p* <.001), living with the father (β = − 0.065, t = − 2.535, *p* =.011), and having a disability (β = 0.090, t = 3.663, *p* <.001) had significant effects on depressive symptoms. Among the different types of victimization, child maltreatment emerged as the most potent predictor (β = 0.201, t = 6.807, *p* <.001), indicating that experiences of maltreatment by caregivers are strongly associated with higher levels of depressive symptoms. Internet victimization also proved to be a significant predictor (β = 0.116, t = 4.028, *p* <.001), underscoring the impact of online harassment and abuse on adolescent mental health. Lastly, both sexual victimization (β = 0.083, t = 2.959, *p* =.003) and peer and sibling victimization (β = 0.080, t = 2.575, *p* =.010) were identified as significant factors, highlighting the broad spectrum of victimization experiences that contribute to depressive symptomatology in this population.

**Table 3:** Multiple Regression Models of Types of Victimization as Predictors of Depressive Symptoms

Predictors	Model 4					Model 5					Model 6				
	B	SE_B_	β	t	*p*	B	SE_B_	Β	t	*p*	B	SE_B_	Β	t	*p*
*Constant*	**7.202**	**0.535**		**13.453**	**0.000**	**6.355**	**0.838**		**7.586**	**0.000**	**6.752**	**0.666**		**10.136**	**0.000**
Gender	**1.737**	**0.273**	**0.160**	**6.353**	**0.000**	**1.997**	**0.383**	**0.184**	**5.215**	**0.000**	**2.009**	**0.383**	**0.185**	**5.251**	**0.000**
Mother	− 0.560	0.465	− 0.030	-1.204	0.229	− 0.068	0.748	− 0.004	− 0.091	0.927	− 0.524	0.469	− 0.028	-1.116	0.265
Father	**− 0.713**	**0.281**	**− 0.065**	**-2.535**	**0.011**	**− 0.958**	**0.409**	**− 0.088**	**-2.342**	**0.019**	**− 0.968**	**0.409**	**− 0.089**	**-2.367**	**0.018**
Siblings	0.015	0.296	0.001	0.050	0.960	0.202	0.433	0.018	0.466	0.641	0.251	0.428	0.022	0.586	0.558
Grandparents	− 0.196	0.372	− 0.013	− 0.525	0.599	0.476	0.503	0.032	0.948	0.343	0.428	0.499	0.029	0.858	0.391
Other relatives	0.077	0.385	0.005	0.200	0.842	0.321	0.608	0.021	0.528	0.597	0.315	0.608	0.021	0.518	0.604
Disability	**1.261**	**0.344**	**0.090**	**3.663**	**0.000**	0.959	0.513	0.068	1.869	0.062	0.981	0.512	0.070	1.914	0.056
CC	0.148	0.102	0.046	1.445	0.149	0.224	0.152	0.070	1.474	0.141	0.219	0.152	0.068	1.446	0.148
CM	**1.121**	**0.165**	**0.201**	**6.807**	**0.000**	**1.489**	**0.230**	**0.267**	**6.473**	**0.000**	**1.472**	**0.229**	**0.264**	**6.429**	**0.000**
PSV	**0.393**	**0.153**	**0.080**	**2.575**	**0.010**	0.322	0.216	0.065	1.490	0.137	0.331	0.216	0.067	1.533	0.125
SV	**0.470**	**0.159**	**0.083**	**2.959**	**0.003**	0.426	0.251	0.076	1.696	0.090	0.429	0.251	0.076	1.708	0.088
WIV	− 0.037	0.100	− 0.011	− 0.364	0.716	− 0.116	0.139	− 0.036	− 0.838	0.402	− 0.114	0.139	− 0.035	− 0.824	0.410
IV	**1.002**	**0.249**	**0.116**	**4.028**	**0.000**	**0.862**	**0.353**	**0.100**	**2.442**	**0.015**	**0.875**	**0.353**	**0.101**	**2.481**	**0.013**
Migrant	–	–	–	–	–	1.389	1.087	0.128	1.278	0.202	0.754	0.722	0.070	1.044	0.297
Gender*Migrant	–	–	–	–	–	− 0.431	0.550	− 0.034	− 0.784	0.433	− 0.437	0.550	− 0.035	− 0.795	0.427
Mother*Migrant	–	–	–	–	–	− 0.752	0.961	− 0.069	− 0.782	0.434	–	–	–	–	–
Father*Migrant	–	–	–	–	–	0.493	0.565	0.040	0.872	0.383	0.496	0.565	0.040	0.877	0.380
Siblings*Migrant	–	–	–	–	–	− 0.332	0.598	− 0.029	− 0.554	0.579	− 0.428	0.585	− 0.037	− 0.732	0.464
Grandparents*Migrant	–	–	–	–	–	**− 1.696**	**0.777**	**− 0.074**	**− 2.184**	**0.029**	**− 1.636**	**0.773**	**− 0.071**	**-2.117**	**0.034**
Other relatives*Migrant	–	–	–	–	–	− 0.503	0.789	− 0.027	− 0.637	0.524	− 0.466	0.788	− 0.025	− 0.592	0.554
Disability*Migrant	–	–	–	–	–	0.559	0.695	0.031	0.805	0.421	0.544	0.694	0.030	0.784	0.433
CC*Migrant	–	–	–	–	–	− 0.099	0.193	− 0.030	− 0.514	0.607	− 0.095	0.193	− 0.029	− 0.493	0.622
CM*Migrant	–	–	–	–	–	**− 0.728**	**0.324**	**− 0.109**	**-2.247**	**0.025**	**− 0.705**	**0.323**	**− 0.106**	**-2.186**	**0.029**
PSV*Migrant	–	–	–	–	–	0.171	0.297	0.029	0.576	0.565	0.161	0.297	0.028	0.544	0.586
SV*Migrant	–	–	–	–	–	0.094	0.324	0.014	0.291	0.771	0.096	0.324	0.014	0.295	0.768
WIV*Migrant	–	–	–	–	–	0.120	0.187	0.037	0.641	0.521	0.116	0.187	0.035	0.617	0.537
IV*Migrant	–	–	–	–	–	0.203	0.497	0.018	0.409	0.683	0.191	0.497	0.016	0.384	0.701
Model Fit			F	**27.084**	**0.000**			F	**13.616**	**0.000**			F	**14.121**	**0.000**
			R^2^	**0.207**				R^2^	**0.216**				R^2^	**0.216**	
			R^2^_adj_	**0.199**				R^2^_adj_	**0.200**				R^2^_adj_	**0.200**	

The values in bold indicate statistically significant values (p <.05)

CC = Conventional Crime; CM = Child Maltreatment; PSV = Peer and Sibling Victimization; SV = Sexual Victimization; WIV = Witnessing and Indirect Victimization; IV = Internet Victimization; β = Standardized Beta

Expanding on this, Model 5 included a migrant status variable and its interaction with other predictors to examine differences between migrant and non-migrant adolescents. The adjusted R² slightly increased to 0.200, signaling a very modest enhancement in the model’s explanatory power with the inclusion of migrant status (F_[27, 1334]_ = 13.616, *p* <.001, D-W = 2.052). In this model, gender (β = 0.184, t = 5.215, *p* <.001), living with the father (β = − 0.088, t = − 2.342, *p* =.019), child maltreatment (β = 0.267, t = 6.473, *p* <.001), and internet victimization (β = 0.100, t = 2.442, *p* =.015) maintained their significance and explanatory direction regarding depressive symptoms. However, having a disability, sexual victimization, and peer victimization lost their significance. This change in significance could be attributed to the introduction of interaction terms in the model, which may have redistributed the explanatory power among the predictors, or it might reflect the complex and multifaceted nature of depressive symptoms, where certain factors become more pronounced or diminish in influence when considered in conjunction with others. Like the findings in previous models on polyvictimization, the interaction between living with grandparents and being a migrant exhibited a significant negative effect on the development of depressive symptoms (β = − 0.074, t = − 2.184, *p* =.029). Yet, the most striking effect was observed in the interaction between child maltreatment and being a migrant, showing a negative effect on depressive symptoms (β = − 0.109, t = 2.247, *p* =.025). In other words, this suggests that the adverse impact of child maltreatment on depressive symptoms might be mitigated on migrants adolescents, potentially indicating a unique resilience or differing coping mechanisms within this group.

Due to potential multicollinearity, particularly with interactions involving living with the mother and migrant status (VIF = 13.184), Model 6 was adjusted by removing these interactions. This model continued to significantly explain the variance in depressive symptoms among adolescents, achieving an adjusted R² of 0.200 (F_[26, 1335]_ = 14.121, *p* <.001, D-W = 2.053). Gender (β = 0.185, t = 5.251, *p* <.001), living with the father (β = − 0.089, t = − 2.367, *p* =.018), and the presence of a disability (β = 0.070, t = 1.914, *p* =.056) remained significant predictors of depressive symptoms. Similarly, child maltreatment (β = 0.264, t = 6.429, *p* <.001) and internet victimization (β = 0.101, t = 2.481, *p* =.013) sustained their significant associations with depressive symptoms, underlining the persistent impact of these experiences on adolescent mental health. Once again, the interaction between living with grandparents and being a migrant (β = − 0.071, t = − 2.117, *p* =.034) suggested a protective effect against depressive symptoms for migrant adolescents. Furthermore, the interaction between child maltreatment and migrant status continued to show a significant negative influence on depressive symptoms (β = − 0.106, t = − 2.186, *p* =.029), indicating that the negative impact of child maltreatment on depressive symptoms might be mitigated for migrant adolescents.

## Discussion

The present study analyzed the relationship between victimization, polyvictimization, and symptoms of depression, comparatively, between Chilean and migrant children and youth born abroad and residing in Chile. The results suggest that migrant children and youths have a higher frequency of victimization for conventional crimes and polyvictimization than their Chilean-born peers. These results concur with previous studies on migrant children and youth, where the character of disadvantaged subjects is highlighted compared to the rest of the population [47, 48]. For Von Hentig [48], immigration entails a regression of personal status due to intense uprooting and a sense of helplessness experienced by these people, placing them in a situation of manifest vulnerability [47]. This is particularly acute when this group is composed of children and youth. Migrants are socially weak victims in their destination country, due to the lack of social ties and support networks that would typically offer assistance in times of need [49]. These conditions place them in vulnerable circumstances to be victims of conventional crimes, such as robberies, assaults, scams and discrimination attacks in the community context [50]. Furthermore, these situations are sometimes aggravated because immigrants do not have sufficient resources to meet their needs for protection and security. This force them to face issues such as housing deprivation or insertion in neighborhoods with high levels of insecurity or criminality [51].

Following the same line, migrant children and youth are more exposed to the accumulative effects of different forms of victimization in several contexts (polyvictimization). This involves sexual victimization, victimization by caregivers, victimization by peers or siblings, indirect victimization, and electronic victimization. This vulnerability is also exacerbated by limited access to safe migration channels, services, and justice. When they arrive in a destination country, they may encounter other difficulties, such as a lack of access to basic services, which makes them highly vulnerable to suffering different forms of violence [34, 35, 52]. Therefore, migrant children experience harsh realities characterized by multiple, intersecting and overlapping problems. The emotional, mental, and physical toll of uncertainty, often harrowing journeys, can undermine their ability to protect themselves, making them even more vulnerable to violence and abuse [52–55]. Furthermore, migrant children already settled in their destination countries suffer the catastrophic consequences of a failure of child protection by international migration-receiving states [56]. In this sense, the results of the study align with similar trends observed in other studies conducted in Chile and various countries, regarding the different forms of victimization experienced by migrant children and adolescents in different contexts [4, 12, 17, 33, 34]. This condition challenges Chile in the implementation of political public policies that socially address the full inclusion of migrant children and adolescents in our society and Latin America [31].

The first hypothesis (H1) was accepted, indicating a positive association between victimization and polyvictimization with depressive symptoms in children and youth, irrespective of their immigration status. This finding aligns with previous research on the topic [57, 58], as all types of individual victimization considered in the present study were associated with depressive symptoms. These data reinforce that early victimization experiences influence mental health problems, specifically the emergency of depression [58, 59]. As Turner et al. [60] stated, experiences of some victimizations are considered a predictor of depression, and any additional victimization increases the severity of depression due to the cumulative risk effect. Specifically, in the current study, we found that child maltreatment, sexual victimization, peer victimization, and electronic victimization were significant predictors of depression. These findings align with previous studies on the subject [13, 14, 36, 61].

In Chile, previous evidence also establishes the relationship between single-type victimizations, polyvictimization, and depression symptoms [37, 62]. However, the findings from the present study should be analyzed with caution, considering depression in Chilean-born children and youth as a complex phenomenon of multicausality. This implies that environmental factors related to victimization and polyvictimization experiences explain only a part of the variance of depression as a mental health problem.

Hypothesis two (H2) was rejected. Despite finding differences between polyvictimization and symptoms of depression between migrant and non-migrant children and youth, the migrant condition or migrant affected by polyvictimization does not have an “additional” effect over the depressive symptoms. Victimization partially accounts for depressive symptoms in both migrant and native children and adolescents.

These findings are contrary to previous studies that mention worse indicators of depression in migrant Children and youth compared to Chilean-born children and youth [63]. However, coincide with other studies that have sought to explore the factors contributing to the positive adaptation of migrant Chileans and youth in the new contexts in which they develop [64, 65].

It is important to mention that childhood depression in Chile is a long-standing and highly prevalent public health problem, which also responds to multiple factors [66]. The country has high rates of childhood depression and suicide [66, 67], and some of the relevant factors that have been considered key are the high rates of violence and mistreatment of children and adolescents in the country [36]. In this context, it has been documented that Chilean children and youth are exposed to high rates of victimization compared to children and adolescents from other cultural contexts [30].

Nevertheless, regarding migrant children and adolescents, and considering the results obtained, it is possible to establish that the problem of depression is a significant problem for children and adolescents regardless of their migratory status. However, it is crucial to analyze the specific results to understand why experiences of victimization/polyvictimization explain depression symptoms in a similar manner for both migrant children and youth as well as Chilean children and youth. In migrant children and adolescents, other risk factors could co-exist that also explain mental health problems (including depression) [63] and that needs to be further explored in future research. Another relevant aspect to consider is related to the adaptive capacity of migrant children in terms of their resilient resources and agency, which could favor their positive adaptation. Both aspects have been described in the literature and could help explain the findings of this study.

The first of these is related to the phenomenon of the “immigrant paradox” [68, 69], in which the adaptation of young migrants is more favorable than expected and, in some cases, better than the adaptation of their non-migrant peers [70] or that of first-generation immigrants [69]. However, the immigrant paradox has not yet been documented in depth with international migrants in Latin America. The second phenomenon that allows us to give a tentative answer to the findings obtained relates to the resilient resources that may be present in young migrants. Thus, some children and youth could have greater social and personal resources, which can have influence and favor their positive adaptation to new environments [71, 72]. According to Sam [73], social influences, acculturation, and preferences of receiving societies contribute significantly to immigrants. Accordingly to this author, societies that value cultural diversity and embrace multicultural openness promote the integration and positive adaptation of migrants. Similarly, receiving societies with more liberal immigration laws that grant additional rights to immigrants contribute to their positive adaptation within these societies [74, 75]. While not as prominent, these characteristics may also be present in Chilean society, which could have facilitated the integration of Latin American migrants in Chile, particularly those who arrived two decades ago.

Another finding of the study is related to the family structure of the adolescents. The results show that living with the father and the presence of grandparents, especially in the case of migrant adolescents, was a protective factor in terms of depressive symptomatology. These results are consistent with previous studies that the family environment may have a fundamental influence on depression [76]. In this case, this research showed family that they may become a protective factor for the depression in migrant adolescents [77, 78]. Gilliom et al. [79], posit that the family can exert behavioral control in adolescents, often reflecting parental care and love, accompanied by positive reinforcement and verbal guidance. This may favor the psychological well-being of adolescents, thus becoming a buffer in the onset and development of depressive symptoms [79, 80].

Alternatively, to the above, another explanation has to do with cultural aspects. Rothenberg et al. [81], states that a culture where family intimacy and control of parental behavior is especially valued, favors adolescents to present fewer problems of internalizing symptomatology. Thus, in cultures where there is a greater emphasis on mutual support and intimacy among the members of the family system, it could be a protective factor against depressive symptoms [78]. These could be characteristics of some cultures from which migrant families come in Chile. It should be noted that these explanations are plausible and should be addressed in future studies that evaluate the perception of adolescents regarding the functioning and quality of relationships in the family system, beyond the structure of the family.

A possible explanation regarding to the child maltreatment in migrant adolescents have an inverse effect on the prediction of depressive symptoms could be also partly related to “immigrant paradox”. In this sense, children and adolescents who experience child maltreatment may develop individual strengths despite their experience of victimization such as coping skills, resilience, self-esteem, as protective factors [82].. Another explanation for this may be through post-traumatic growth, a concept that alludes to the perception of positive changes experienced as a result of facing traumatic experiences, favoring the development of a better level of psychological functioning than that experienced before the event [83–85]. Individuals who experience growth exhibit significant positive changes and an enhanced sense of life that emerge as a result of coping with a traumatic event [86].

In addition, migrant children and adolescents may have experienced multiple forms of victimization in their country of origin, including political violence, gang violence, mistreatment, abuse and neglect [86, 87]. In this context, migration may be experienced as a form of protection against the violence they have experienced before arriving in Chile [35], thereby decreasing depressive symptomatology.

### Limitations

Despite the findings described, the present study has some limitations. The first is related to the methodological aspects of the cross-sectional-correlational design used, making it difficult to make causal interpretations. Additionally, the evidence provided on the variables studied is preliminary. Another limitation is the impossibility of determining an apparent temporal sequence between the variables because the measurement of both types of variables is done simultaneously [88]. Regarding the characteristics of the instrument, these may represent another limitation of the study since the questionnaire used could affect social desirability or acquiescence [89], which could lead to some bias in the responses. However, although these biases could be present, they are insufficient to invalidate retrospective research on childhood victimization experiences. On one hand, future research in this area could incorporate qualitative techniques for data collection, such as interviews, focus groups, lifelines, etc., to reduce reporting bias. On the other hand, it is important to note that the study included the variables sex, age, and grade level in the multivariate regression analysis, as a way of controlling for possible confounding bias, and no effects of these variables were observed in the results of the study. It is also important to point out that in the National Polyvictimization Survey, types of victimizations were analyzed based on the occurrence of the events, not considering other variables, such as the duration, frequency, or severity of the events, or the type of aggressor. This could be confounding variables in the results. For this reason, it is proposed that future studies could incorporate these variables as a way of controlling the possible influences they may have on the results of the study.

Finally, this study showed the experiences of victimization and their connection with depressive symptoms in migrant children and youth within the Chilean educational system. Yet, the relationship between victimization and depressive symptomatology in migrant children and youth who are refugees or outside the educational system is still unknown. Nevertheless, this study addresses a significant limitation often seen in prior research in this field, by adopting an integral approach to evaluate polyvictimization and its associations with mental health issues in immigrants, children and youth.

### Practical implications

The results of this study have practical implications for the analysis of migration in the Chilean context and for promoting actions to support international migration between Andean countries, especially for vulnerable young people. The findings of this research show that migrant children and youth are exposed to multiple types of violence in different contexts. This information could be used as input for the Chilean State concerning the situations of lack of protection in vulnerable migrant children and youth. Additionally, organizations such as the IOM (International Office for Migration) or municipalities working in the territories and prevent community violence can use these findings. Additionally, the results can also be used by the programs of the Ministry of Health of Chile to emphasize the migrant children and youth’s mental health needs and make the victimization/polyvictimization visible and a risk factor in their clinical interventions. Along the same lines, the Ministry of Education is to develop actions to prevent violence and promote school connection, social support, and positive adaptation of migrant children and youth in Chile.

## Conclusion

The findings of this study lead us to conclude that migrant children and youth in Chile are subject to a wide range of victimizations, with victimization by conventional crimes and polyvictimization being notably prevalent. Furthermore, victimization and polyvictimization have a significant effect on depression across both migrant and Chilean-born children and adolescents, underscoring a universal challenge transcending migration status. A particularly compelling finding is the observed interaction between child maltreatment and migrant status, which surprisingly indicates a mitigated impact of maltreatment on depressive symptoms among migrant adolescents, suggesting that migrant adolescents might possess unique resilience or employ differing coping mechanisms in the face of adversity, a testament to their adaptability and strength. Future research should focus on elucidating these protective mechanisms and exploring how policy interventions can best leverage the adaptive capacities of migrant children and adolescents, ensuring their successful integration and well-being in host countries.

## Data Availability

This data is part of a secondary study. The data are for the universities from Chile transferred from the Government of Chile. However, the data are anonymous, and it is not possible to identify the participants.

## Footnotes

1 In the Chilean educational system, ‘first year of high school’ corresponds to the ninth grade, ‘second year of high school’ to the tenth grade, and ‘third year of high school’ to the eleventh grade in the U.S. educational system.

2 A detailed correlation matrix is available upon direct request to the corresponding author.
